# Epidemiological consequences of immune sensitisation by pre-exposure to vector saliva

**DOI:** 10.1371/journal.pntd.0005956

**Published:** 2017-10-09

**Authors:** Tsukushi Kamiya, Megan A. Greischar, Nicole Mideo

**Affiliations:** Department of Ecology & Evolutionary Biology, University of Toronto, Toronto, Ontario, Canada; Fundacao Oswaldo Cruz, BRAZIL

## Abstract

Blood-feeding arthropods—like mosquitoes, sand flies, and ticks—transmit many diseases that impose serious public health and economic burdens. When a blood-feeding arthropod bites a mammal, it injects saliva containing immunogenic compounds that facilitate feeding. Evidence from *Leishmania*, *Plasmodium* and arboviral infections suggests that the immune responses elicited by pre-exposure to arthropod saliva can alter disease progression if the host later becomes infected. Such pre-sensitisation of host immunity has been reported to both exacerbate and limit infection symptoms, depending on the system in question, with potential implications for recovery. To explore if and how immune pre-sensitisation alters the effects of vector control, we develop a general model of vector-borne disease. We show that the abundance of pre-sensitised infected hosts should increase when control efforts moderately increase vector mortality rates. If immune pre-sensitisation leads to more rapid clearance of infection, increasing vector mortality rates may achieve greater than expected disease control. However, when immune pre-sensitisation prolongs the duration of infection, e.g., through mildly symptomatic cases for which treatment is unlikely to be sought, vector control can actually increase the total number of infected hosts. The rising infections may go unnoticed unless active surveillance methods are used to detect such sub-clinical individuals, who could provide long-lasting reservoirs for transmission and suffer long-term health consequences of those sub-clinical infections. Sensitivity analysis suggests that these negative consequences could be mitigated through integrated vector management. While the effect of saliva pre-exposure on acute symptoms is well-studied for leishmaniasis, the immunological and clinical consequences are largely uncharted for other vector-parasite-host combinations. We find a large range of plausible epidemiological outcomes, positive and negative for public health, underscoring the need to quantify how immune pre-sensitisation modulates recovery and transmission rates in vector-borne diseases.

## Author summary

Many diseases of health and economic importance are transmitted by arthropod vectors, like mosquitoes, sand flies, and ticks. When a blood-feeding arthropod bites a mammal, it injects saliva containing compounds that facilitate feeding. The immune responses elicited by previous exposure to vector saliva can alter disease severity if the host later becomes infected. Such pre-sensitisation of host immunity has been linked to either exacerbation or mitigation of symptoms in a number of disease systems. We develop a general model of vector-borne disease to examine how vector control efforts alter the frequency of immune pre-sensitisation and thus change the epidemiological impact of control. We show that the abundance of pre-sensitised infected hosts should increase when control efforts moderately increase vector mortality rates. When immune pre-sensitisation leads to longer infections—by generating sub-clinical cases for which treatment is not rapidly sought—killing vectors can lead to unexpected increases in the number of infected hosts. The rising case burden may go unnoticed unless sub-clinical individuals are tested for infection. Conversely, if immune pre-sensitisation leads to more rapid clearance of infection, increasing vector mortality rates may achieve greater than expected disease control. Our findings highlight the need to quantify how immune pre-sensitisation modulates clinical outcomes and parasite transmission in humans.

## Introduction

When a mammal is bitten by a blood feeding arthropod, it is injected with vasodilatory and immunomodulatory compounds in the arthropod’s saliva that facilitate feeding [1–4]. The mammalian host is not a passive recipient in this interaction, but rather mounts a variety of immune responses [5]. Local immune responses to an arthropod bite include inflammation, production of anti-salivary protein antibody and recruitment of immune cells to the skin. These same arthropods can be vectors of important parasites and a recent focus of research has been elucidating the influence of salivary proteins on transmission of a wide array of diseases, including those caused by protozoan parasites such as *Plasmodium* and *Leishmania* and arboviruses such as dengue and West Nile virus [5–10]. In a majority of lab experiments, parasites that are co-inoculated with vector saliva or salivary proteins show higher infection success than when parasites are injected alone [11, 12]. Co-inoculation can benefit parasite establishment through saliva proteins that modulate host immune responses (e.g., downregulating particularly harmful pathways or upregulating pathways that will inhibit parasite-specific responses) and can lead to the recruitment of immune cells that those parasites exploit for replication [5, 7, 8, 12].

In contrast, immune responses elicited by the bite of an uninfected vector appear to have diverse effects on the outcome of disease manifestation if a host later becomes infected. In *Leishmania*, from which the majority of empirical evidence is available, experimental rodent infections have demonstrated that prior exposure to sand fly saliva partially protects against the symptoms of a subsequent infection [13–17]. A recent meta-analysis found a significant reduction in *Leishmania* lesion development and a marginally significant reduction in parasite load due to pre-exposure to sand fly saliva [11]. Such protective properties of exposure to uninfected saliva (“pre-sensitisation”) have garnered enthusiasm for the development of anti-leishmaniasis vaccines using sand fly saliva proteins [18]. For malaria, prior exposure to mosquito saliva has been shown to reduce *Plasmodium* burden in the liver- and blood-stage [19] while no pre-sensitisation effect was found for the infectivity of sporozoites (i.e., the stage that is transferred from vector to host) [9]. In West Nile virus, empirical evidence for the role of pre-exposure to mosquito saliva is mixed [10, 20]. The immunological mechanisms behind any protective effects remain an open question [3], but may include direct effects on parasites (e.g., polarisation of the immune response towards microbial killing [5]) or indirect effects, like neutralising saliva proteins that would otherwise facilitate parasite proliferation [5].

Classic epidemiological models tend to ignore the effects of immune pre-sensitisation. These models (e.g. [21–23]) predict that heightened adult vector mortality can effectively control a vector-borne disease by reducing vector abundance, the number of bites per vector, and the probability of surviving the extrinsic incubation period (i.e., the time it takes for an exposed vector to become infectious; reviewed in [24, 25]). These theoretical predictions encouraged the World Health Organization (WHO) to carry out a worldwide insecticide spraying campaign against the mosquito vectors of malaria parasites, with successful elimination of the disease reported in many countries by the late 1970s [26]. Insecticide spraying remains a frontline prevention and control strategy against malaria and many other vector-borne diseases, including dengue and leishmaniasis [26–28]. While insecticides have generally proven effective in reducing the incidence of malaria [29], there is considerable heterogeneity in the efficacy of spraying reported: interventions that target adult vector survival have failed to reduce the number of infections in some host populations [30–32]. These outcomes have been attributed to a number of factors ranging from insecticide resistance, sublethal exposure, behavioural alterations by arthropod vectors to avoid insecticides, the presence of a non-human reservoir, heterogeneity in vector life-history traits, and spatial and temporal variation in host and vector populations [31–35], though the relative role of each of these factors is unknown.

Here we develop mathematical models to determine the potential for pre-exposure to vector saliva to affect between-host infection dynamics and modulate the consequences of interventions that target vectors. Where vector-borne diseases are endemic, a significant proportion of individuals are likely pre-sensitised by vector saliva; in one sample of individuals from Mali, 23% demonstrated a robust immune response against sand fly salivary molecules [36]. Our work shows that the interplay between vector saliva and host immunity can produce a variety of epidemiological outcomes, depending on the effect of pre-sensitisation on the duration of infection.

## Materials and methods

### Model

**Fig 1:**
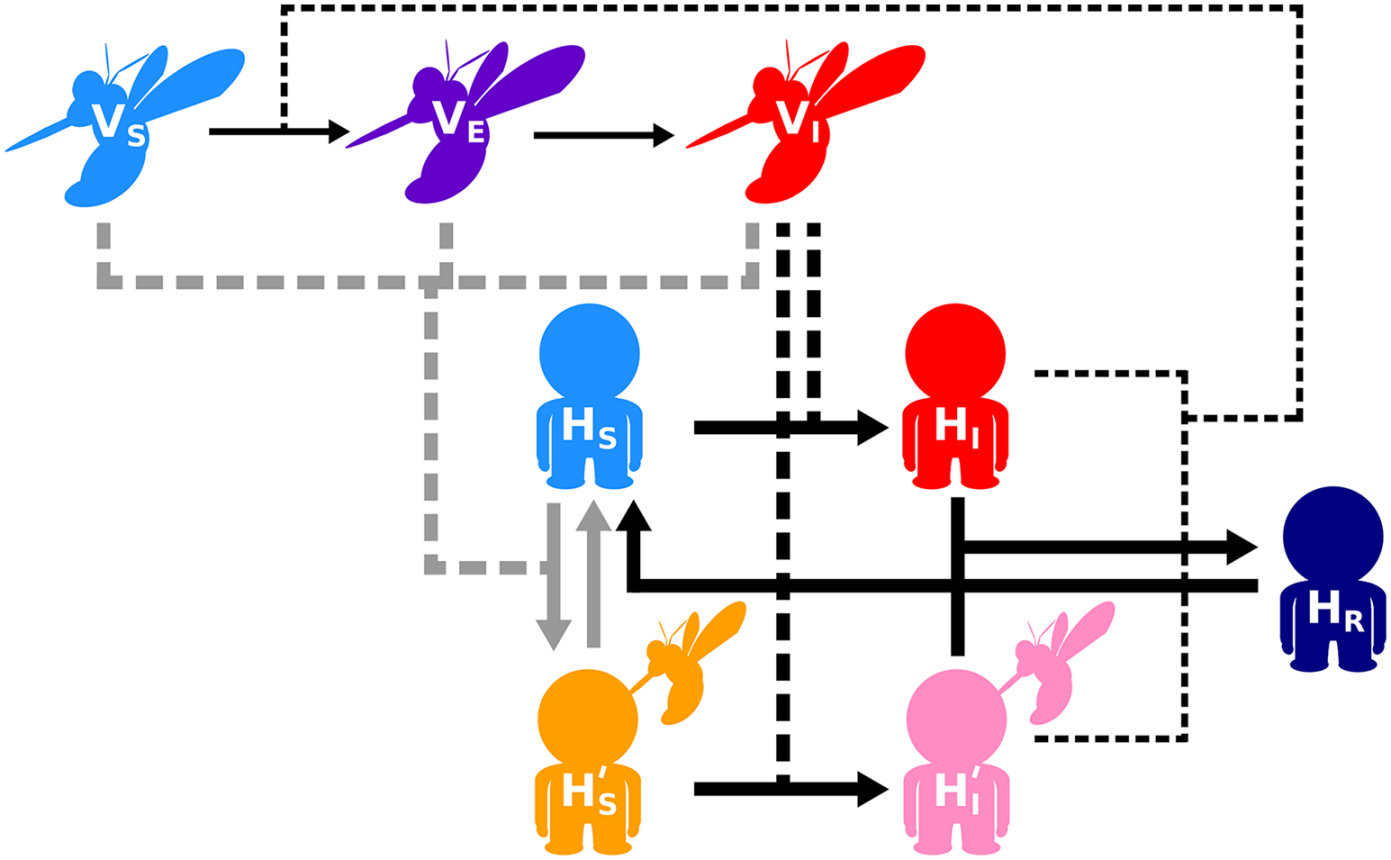
Schematic of vector-borne disease dynamics model when host immunity can be pre-sensitised through vector saliva pre-exposure. Susceptible hosts (*H_S_*) may become pre-sensitised ($H_{S}^{\prime }$) when bitten by either a susceptible vector (*V_S_*), an exposed but non-infectious vector (*V_E_*), or an infectious vector (*V_I_*) if parasite transmission is unsuccessful. Susceptible hosts that are pre-exposed to vector saliva remain sensitised until the protective status is lost over time. Susceptible pre-sensitised and naïve hosts ($H_{S}^{\prime }$ and *H_S_* respectively) can become infected ($H_{I}^{\prime }$ and *H_I_* respectively) when bitten by an infectious vector. Upon recovering from infection, the host gains immunity against future infections (*H_R_*), but that immunity wanes over time. Further details can be found in Methods. Infection routes of hosts are shown in thick black; pre-sensitisation routes in grey; infection of vectors in thin black. The movement between classes is shown in solid lines and the interactions that lead to that movement are shown by dashed lines.

We modelled vector-borne disease dynamics as a set of ordinary differential equations (ODE), and alternatively, as a set of delay differential equations (DDE) that track the change in the abundance of three vector (susceptible, exposed and infectious) and five host (naïve susceptible, pre-sensitised susceptible, naïve infected, pre-sensitised infected and recovered) classes. We first describe the ODE formulation as follows (Fig 1; Eqs 1–8):

$$\begin{array}{ccc} \frac{dV_{S}\left(t\right)}{dt} & = & \phi_{V}-\left(\mu_{V}+r\left(T_{VH}H_{I}\left(t\right)+T_{VH^{\prime }}H_{I}^{\prime }\left(t\right)\right)\right)V_{S}\left(t\right) \end{array}$$ (1)

$$\begin{array}{ccc} \frac{dV_{E}\left(t\right)}{dt} & = & r\left(T_{VH}H_{I}\left(t\right)+T_{VH^{\prime }}H_{I}^{\prime }\left(t\right)\right)V_{S}\left(t\right)-\left(\mu_{V}+\sigma_{V}\right)V_{E}\left(t\right) \end{array}$$ (2)

$$\begin{array}{ccc} \frac{dV_{I}\left(t\right)}{dt} & = & \sigma_{V}V_{E}\left(t\right)-\mu_{V}V_{I}\left(t\right) \end{array}$$ (3)

$$\begin{array}{ccc} \frac{dH_{S}\left(t\right)}{dt} & = & -\left(rP_{HV}\left(V_{S}\left(t\right)+V_{E}\left(t\right)+\left(1-T_{HV}\right)V_{I}\left(t\right)\right)+rT_{HV}V_{I}\left(t\right)\right)H_{S}\left(t\right)\\ & & +\theta_{H^{\prime }}H_{S}^{\prime }\left(t\right)+\tau_{H}H_{R}\left(t\right) \end{array}$$ (4)

$$\begin{array}{ccc} \frac{dH_{S}^{\prime }\left(t\right)}{dt} & = & \left(rP_{HV}\left(V_{S}\left(t\right)+V_{E}\left(t\right)+\left(1-T_{HV}\right)V_{I}\left(t\right)\right)\right)H_{S}\left(t\right)\\ & & -\left(rT_{H^{\prime }V}V_{I}\left(t\right)+\theta_{H^{\prime }}\right)H_{S}^{\prime }\left(t\right) \end{array}$$ (5)

$$\begin{array}{ccc} \frac{dH_{I}\left(t\right)}{dt} & = & rT_{HV}V_{I}\left(t\right)H_{S}\left(t\right)-\gamma_{H}H_{I}\left(t\right) \end{array}$$ (6)

$$\begin{array}{ccc} \frac{dH_{I}^{\prime }\left(t\right)}{dt} & = & rT_{H^{\prime }V}V_{I}\left(t\right)H_{S}^{\prime }\left(t\right)-\gamma_{H^{\prime }}H_{I}^{\prime }\left(t\right) \end{array}$$ (7)

$$\begin{array}{ccc} \frac{dH_{R}\left(t\right)}{dt} & = & \gamma_{H}H_{I}\left(t\right)+\gamma_{H^{\prime }}H_{I}^{\prime }\left(t\right)-\tau_{H}H_{R}\left(t\right). \end{array}$$ (8)

While the process of vector input into the population is complex and likely important for predicting disease dynamics, data on the potentially density-dependent processes in vector population dynamics are scarce [37]. Therefore, we assumed that susceptible vectors (*V*_*S*_) are born at a constant rate, *ϕ*_*V*_. We also assumed that all vectors experience the same mortality rate, *μ*_*V*_, regardless of their infection status. In our model, vectors are equally likely to bite a host of any class, so hosts get bitten by a given vector at the rate *r*, which is calculated as the per vector biting rate, *b*, divided by the total host population size, *H*_*T*_ (where $H_{T}=H_{S}+H_{S}^{\prime }+H_{I}+H_{I}^{\prime }+H_{R}$). This formalisation makes transmission frequency-dependent [38]. A susceptible vector becomes exposed (*V*_*E*_) to parasites when it bites a naïve infected host (*H*_*I*_) or a pre-sensitised infected host ($H_{I}^{\prime }$) with the probability *T*_*VH*_ or *T*_*VH*′_, respectively. In this ODE model, exposed vectors are assumed to become infectious (*V*_*I*_) at a constant incubation rate, *σ*_*V*_, which is the inverse of the mean extrinsic incubation period (EIP, 1/*σ*_*V*_). Thus, EIP follows an exponential distribution with large variance ($\sigma_{V}^{-2}$), leading to an implicit assumption that the parasite can complete development in the vector at any time, even immediately after a vector becomes exposed. Empirical estimates of the variability in the EIP are rare, but it is known to vary on the order of days in dengue (approx. 5-33 days at 25°C [39]) and malaria (approx. 5-14 days at 24°C [40]) and that variation is thought to influence disease transmission [40]. To examine whether our results are sensitive to the assumption of large variability in the duration of the EIP, we also formulated the model as a system of delay differential equations (DDEs), which assumes the opposite extreme of no variation in EIP: all exposed vectors require exactly the length of the mean EIP, or 1/*σ*_*V*_ days to become infectious, though some vectors may not survive that period.

The DDE formulation differs from the ODE counterpart in two equations (ODE: Eqs 2 & 3; DDE: Eqs 9 & 10), which describe the dynamics of exposed and infectious vectors, respectively:

$$\begin{array}{ccc} \frac{dV_{E}\left(t\right)}{dt} & = & r\left(T_{VH}H_{I}\left(t\right)+T_{VH^{\prime }}H_{I}^{\prime }\left(t\right)\right)V_{S}\left(t\right)-\mu_{V}V_{E}\left(t\right)\\ & & -r\left(T_{VH}H_{I}\left(t-EIP\right)+T_{VH^{\prime }}H_{I}^{\prime }\left(t-EIP\right)\right)V_{S}\left(t-EIP\right)e^{-\mu_{V}EIP} \end{array}$$ (9)

$$\begin{array}{ccc} \frac{dV_{I}\left(t\right)}{dt} & = & r\left(T_{VH}H_{I}\left(t-EIP\right)+T_{VH^{\prime }}H_{I}^{\prime }\left(t-EIP\right)\right)V_{S}\left(t-EIP\right)e^{-\mu_{V}EIP}\\ & & -\mu_{V}V_{I}\left(t\right) \end{array}$$ (10)

where *e*^−*μ*_*V*_*EIP*^ is the probability that an exposed vector survives the length of the extrinsic incubation period (EIP, i.e., 1/*σ*) to become infectious. The simulation starts with a population of susceptible vectors and one infectious vector; there are no exposed vectors initially, so *e*^−*μ*_*V*_*EIP*^ needs only be defined for *t* > 0. Note that this DDE system can be written in terms of only *dV*_*S*_ and *dV*_*I*_, but we retain an exposed vector equation for clarity and ease of comparison with the ODE model.

As in most models of vector-borne diseases (reviewed in [25]), we kept the host population size constant by ignoring host birth and death. The assumption of constant population size is reasonable over the time scale of vector control. Note that our analyses focus on equilibrium conditions of the model (see below). As such, we refer to the equilibrium quantity of a given host class as abundance, number, or cases throughout the manuscript (e.g., abundance of pre-sensitised infected hosts); however, changes in these quantities are concomitant with changes in the proportion or prevalence of the host class as the total host population size is held constant. A susceptible host becomes pre-exposed to vector saliva and pre-sensitised ($H_{S}^{\prime }$) when bitten by a susceptible or an exposed vector (*V*_*S*_ and *V*_*E*_, respectively) with the probability *P*_*HV*_, or by an infectious vector (*V*_*I*_) with the probability *P*_*HV*_(1 − *T*_*HV*_), i.e., when parasite transmission fails upon contact but pre-sensitisation is successful. Alternatively, a susceptible host becomes infected (*H*_*I*_) when bitten by an infectious vector with the probability *T*_*HV*_. Pre-sensitised susceptible hosts lose their protected status at a rate *θ*_*H*′_. Once infected, naïve infected hosts recover from infection at a rate *γ*_*H*_. After a host is pre-sensitised, a bite from an infectious vector creates a pre-sensitised infected host, $H_{I}^{\prime }$ with the probability *T*_*H*′ *V*_. As a host recovers from infection (*H*_*R*_), it becomes temporarily immune to future infections until acquired immunity wanes, which occurs at a rate *τ*_*H*_.

We focus primarily on the effect of vector saliva pre-exposure on the duration of infection by assuming that pre-sensitised infected hosts recover at a unique rate, *γ*_*H*′_. The available data indicate that immune pre-sensitisation through pre-exposure to vector saliva has a likely role in altering parasite growth and density [10, 11, 19, 20, 41]. This could lead, respectively, to reduced or amplified symptoms during a future infection, with knock on consequences for the duration of that infection. For example, if patients with more severe symptoms are more likely to seek treatment, their infections may be shortened by clinical interventions. Therefore, if pre-sensitisation to vector saliva leads to exacerbation of disease symptoms, then this could lead to shorter infections. A similar outcome might be expected if pre-sensitisation leads to increased resistance of the host. Alternatively, disease mitigation through pre-sensitisation may also increase the duration of infection if pre-exposure to vector saliva only provides partial resistance, i.e., parasite growth is hindered, but infections are not cleared—a scenario that is consistent with a meta-analysis on *Leishmania* [11] and some studies on *Plasmodium* [19] and West Nile virus [10]. If pre-sensitised infected patients experience less severe symptoms due to lower parasite burdens, they may also be less likely to seek clinical treatment, thereby prolonging the time to recovery. We also investigated the effect of vector saliva pre-exposure on susceptibility, *T*_*H*′ *V*_, and infectiousness, *T*_*VH*′_ as part of a sensitivity analysis (see Analysis for details). While a reduction in infection-induced host mortality is another potential consequence of immune pre-sensitisation, a meaningful interpretation of such an effect would rely on understanding how host death affects the recruitment of susceptible hosts, a complication outside the scope of the present study.

### Analysis

First, using the ODE model, we derived the abundance of each vector class at quasi-equilibrium to examine the effect of control measures on vector demography. The quasi-equilibrium approach assumes that the lifespan of a vector is much shorter than that of a host so that the vector population quickly reaches a steady state [38]. Mathematically, this means that the rates of change of vector populations, i.e., $\frac{dV_{S}\left(t\right)}{dt}$, $\frac{dV_{E}\left(t\right)}{dt}$ and $\frac{dV_{I}\left(t\right)}{dt}$ (Eqs 1–3), are set to zero and by solving for *V*_*S*_, *V*_*E*_ and *V*_*I*_, we find the quasi-equilibrium vector abundances:

$$\begin{array}{ccc} \hat{V_{S}} & = & \frac{\phi_{V}}{r\left(H_{I}T_{VH}+H_{I}^{\prime }T_{VH^{\prime }}\right)+\mu_{V}}, \end{array}$$ (11)

$$\begin{array}{ccc} \hat{V_{E}} & = & \hat{V_{S}}\frac{r(H_{I}T_{VH}+H_{I}^{\prime }T_{VH^{\prime }})}{\mu_{V}+\sigma_{V}}, \end{array}$$ (12)

$$\begin{array}{ccc} \hat{V_{I}} & = & \hat{V_{E}}\frac{\sigma_{V}}{\mu_{V}}. \end{array}$$ (13)

Second, we numerically simulated both the ODE and DDE model (Eqs 1–8 & 9 and 10) to a stable equilibrium and investigated how the demographic shift in the vector population driven by increased vector mortality affects the abundance of hosts in different classes, in the presence and absence of pre-sensitisation effects of pre-exposure to vector saliva. We performed numerical simulations in *R* Version 3.2.4 [42], using the package *deSolve* [43] to solve for a steady state. The stability of steady states in the ODE model was assessed using the package *rootSolve* [44] while we simulated the DDE model forward in time until the derivatives approached zero with a threshold of 10^−4^. The simulations were initialised with disease-free equilibrium conditions for susceptible vectors and hosts (S1 Appendix) and one infected vector (*V*_*I*_(0) = 1). Whenever we simulated the dynamics in the presence of an intervention targeting vector survival, vector mortality was elevated from the onset of a disease outbreak, mimicking, for example, indoor residual spraying regimes. Parameter values used in our simulations are listed in Table 1. Where possible, default parameter values were chosen from within the range of parameters explored in previous iterations of the Ross-McDonald model of leishmaniasis and malaria dynamics (reviewed in [25, 45]); a wider range of values were explored for parameters describing the process of saliva immune pre-sensitisation due to a paucity of estimates. We also investigated the short term infection dynamics by analysing the effect of saliva pre-exposure on *R*_0_, which characteristically describes the early infection dynamics (S1 Appendix) and by simulating the transient infection dynamics in the presence and absence of a control (S2 Appendix). Finally, using the ODE model, we graphically explored parameter sensitivity of the key findings to identify factors that influence the interaction between vector saliva pre-sensitisation and interventions targeting vector survival (S3 Appendix).

**Table 1:** Model parameters and their values (defaults and ranges explored for parameter sensitivity are listed). Rates are in units of per day unless otherwise indicated.

Symbol	Description	Default (Range)
*ϕ*_*V*_	Vector birth rate	1500 (100, 5000)
*μ*_*V*_	Vector mortality rate	14^−1^ (14^−1^, 14^−1^ + 2.5^−1^)
*r*	Rate at which a host gets bitten by a vector	$\frac{b}{H_{T}}$
*b*	Biting rate per vector	0.15 (0.05, 0.5)
*σ*_*V*_	Parasite incubation rate in vector (1/EIP)	14^−1^ (30^−1^, 2^−1^)
*P*_*HV*_	Pre-sensitisation probability upon contact	0.1 (0, 1)
*H*_*T*_	Total number of hosts	$H_{S}+H_{S}^{\prime }+H_{I}+H_{I}^{\prime }+H_{R}$, 1000
*θ*_*H*′_	Rate of loss of immune pre-sensitisation through saliva pre-exposure effect	0 (0, 14^−1^)
*T*_*HV*_	Transmission probability from vector to naïve host	0.5
*T*_*H*′*V*_	Transmission probability from vector to pre-sensitised host	0.5 (0.05, 0.5)
*T*_*VH*_	Transmission probability from naïve host to vector	0.5
*T*_*VH*′_	Transmission probability from pre-sensitised host to vector	0.5 (0.05, 0.5)
*γ*_*H*_	Recovery rate with acquired immunity of naïve hosts	60^−1^ (150^−1^, 20^−1^)
*γ*_*H*′_	Recovery rate with acquired immunity of pre-sensitised hosts	$\frac{\gamma_{H}}{5}$ ($\frac{\gamma_{H}}{5}$, 2*γ*_*H*_)
*τ*_*H*_	Rate of loss of acquired immunity	2 years^−1^ (10 years^−1^, $\frac{1}{2}\;{\text{year}}^{-1}$)

## Results

### Interventions targeting vector survival facilitate immune pre-sensitisation through pre-exposure to vector saliva

**Fig 2:**
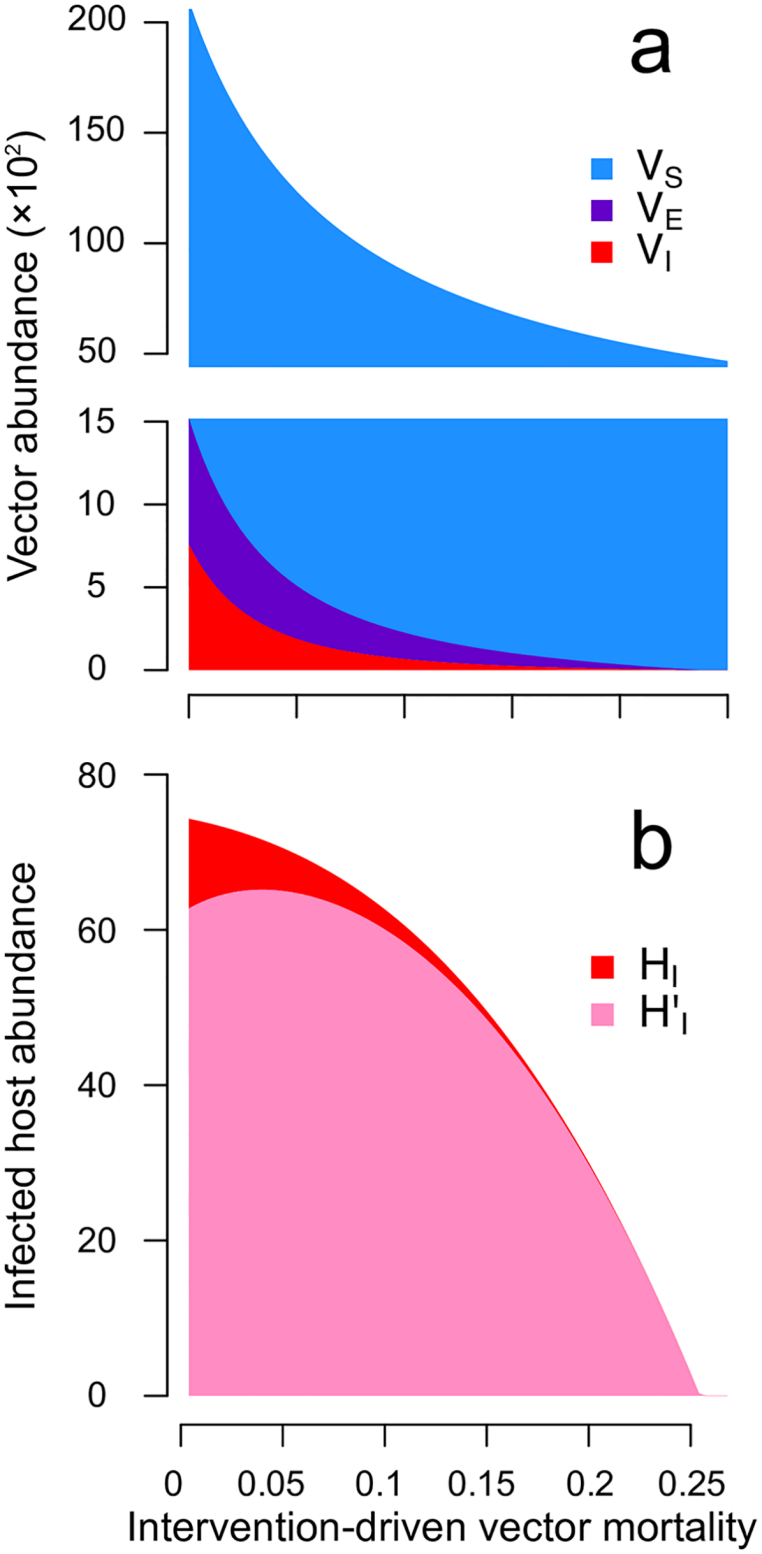
Interventions targeting vector survival, such as insecticide spraying, increase the likelihood of immune pre-sensitisation through pre-exposure to vector saliva. Shown are the equilibrium abundances in the ODE model of (a) susceptible (*V_S_*; blue), exposed (*V_E_*; purple) and infectious (*V_I_*; red) vectors, and (b) infected hosts that are not pre-exposed (*H_I_*; red) and that are pre-exposed ($H_{I}^{\prime }$; pink) to vector saliva. Here, the x-axis is the daily rate of vector mortality imposed by vector control. Pre-sensitised and naïve infected hosts are assumed to have identical recovery rates (*γ_H_* = *γ_H′_* = 60^−1^ per day) and transmission probabilities (*T_HV_* = *T_H′V_* = *T_VH_* = *T_VH′_* = 0.5). Note that the force of infection from vectors, *rT_HV_V_I_*, is proportional to the abundance of infectious vectors, and the rate of immune pre-sensitisation through vector saliva pre-exposure, *rP_HV_*(*V_S_* + *V_E_* + (1 − *T_HV_*)*V_I_*), is roughly proportional to the abundance of susceptible vectors (notice that *V_S_* is at least one order of magnitude larger than *V_E_* or *V_I_*).

Before making any assumptions about the immunological consequences of pre-sensitisation through pre-exposure to vector saliva, we first assume that there are none: pre-sensitised and naïve infected hosts are assumed to have the same rates of recovery and probabilities of onward transmission. From quasi-equilibrium conditions (Eqs 11–13), we can infer the effect of increased vector mortality on the abundance of vectors in different classes, and the subsequent influence on the abundance of infected hosts that are pre-exposed to vector saliva. As expected from the classical Ross-MacDonald model (reviewed in [24, 25]), our model shows that heightened vector mortality incurs multiplicative effects on parasite transmission in the vector population. First, vector abundance declines with vector mortality (Eq 11). Second, the number of times a vector bites during its lifetime is a function of its lifespan, so increased vector mortality reduces the likelihood that a vector bites an infected host and becomes exposed to a parasite (Eq 11). Third, a parasite must survive the extrinsic incubation period in the vector in order for the exposed vector to become infectious and as vector mortality increases, exposed vectors are less likely to survive that period (Eq 12). Additionally, once becoming infectious, vectors are shorter living (Eq 13). Taken together, an increase in vector mortality reduces the total vector abundance, and increases the ratio of non-infectious to infectious vectors by disproportionately reducing the abundance of infectious vectors (Fig 2a).

The impact of control measures on the host population is more nuanced. First, as expected, the reduction in the total vector abundance reduces the rate of contact between hosts and vectors, leaving more hosts in the naïve susceptible class. Second, the processes of infection and saliva pre-sensitisation ‘compete’ for the common resource, naïve susceptible hosts. Thus, the increased ratio of non-infectious to infectious vectors increases the likelihood of vector saliva pre-sensitisation over that of infection. Together with the increase in the number of susceptible hosts, the increasing likelihood of pre-sensitisation increases the abundance of pre-sensitised susceptible hosts as control reduces vector survival. As intuition would suggest, the force of infection from vectors monotonically decreases with the intensity of vector control (Fig 2a). However, over a range of vector mortality values, the increasing availability of pre-sensitised susceptible hosts outweighs the decreasing force of infection from vectors. Consequently, when the increase in vector mortality is moderate, the number of pre-sensitised infected hosts can actually increase ($H_{I}^{\prime }$, Fig 2b; pink). More generally, vector control increases the number of hosts that become pre-exposed to vector saliva prior to an infectious bite. Thus, the proportion of infected hosts that are pre-sensitised always increases with heightened vector mortality (Fig 2b), assuming that the probability of transmission from the vector to host is unaffected by the saliva pre-exposure. In summary, without assuming any effect of vector-saliva pre-exposure on host recovery rate, the overall infection abundance ($H_{I}+H_{I}^{\prime }$) always declines with increasing vector mortality when facing vector control. Importantly, even while the total abundance of infected hosts declines, the number and proportion of infected hosts that are pre-exposed to vector saliva can increase due to the increased availability of susceptible hosts that are pre-exposed to vector saliva.

### A moderate increase in vector mortality can elevate infection cases

**Fig 3:**
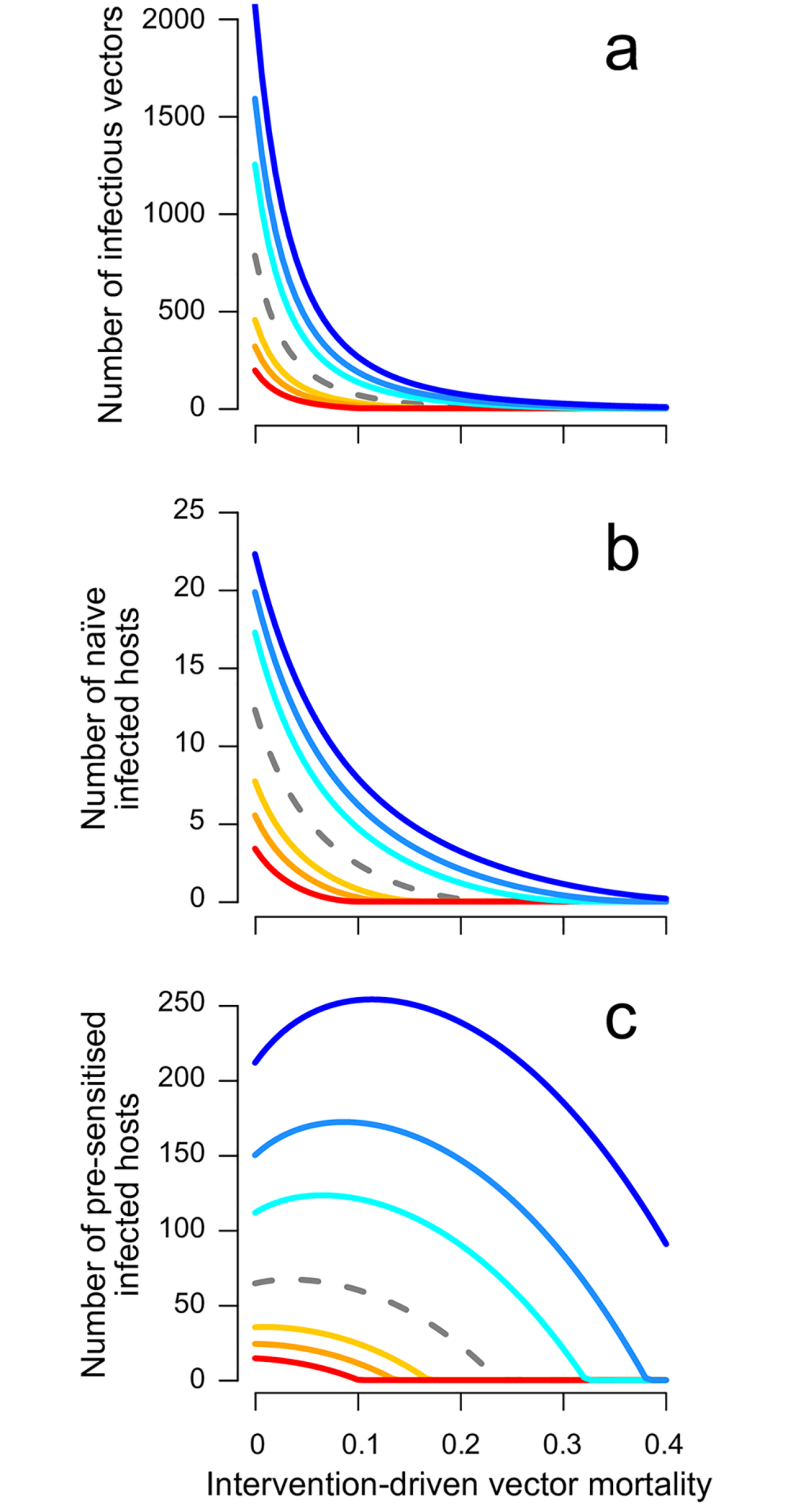
Increasing vector mortality can elevate pre-sensitised and total infection cases when pre-exposure to vector saliva prolongs the time to recovery. Shown are (a) the abundance of infectious vectors, (b) naïve infected hosts, and (c) pre-sensitised infected hosts in the ODE model. The total infection cases in the host population (the sum of b and c) can increase with vector mortality, since pre-sensitised infections are more abundant (note the difference in scale between b and c). The dashed grey line shows the result when pre-exposure has no effect (i.e. *γ*_H′_ = *γ_H_*). Shown in cool colours are the results when pre-sensitisation causes decreased recovery rates with the strength of pre-sensitisation reflected in the intensity of blue: recovery rates of pre-sensitised infected hosts equaling $\frac{1}{2}$, $\frac{1}{3}$, and $\frac{1}{5}$ *th* of the recovery rate of naïve infected hosts. Conversely, the effects of pre-exposure as increased recovery rate are shown in warm colours reflecting 2, 3, and 5 times the recovery rate of naïve infected hosts.

When pre-exposure to vector saliva mitigates disease manifestation and prolongs the time until recovery through clinical intervention, we find that, in the short term, vector control suppresses the peak number of infections in both host and vector populations and slows the spread of infection (S2 Appendix). At equilibrium, increased vector mortality reduces the abundance of infectious vectors (Fig 3a) and the abundance of naïve infected hosts (i.e., those assumed to show clinical symptoms, Fig 3b). The previous section demonstrated that vector control can increase the abundance of pre-sensitised infected hosts (Fig 2b), an increase that is amplified when pre-sensitisation results in longer durations of infection (Fig 3c; cool colours).

**Fig 4:**
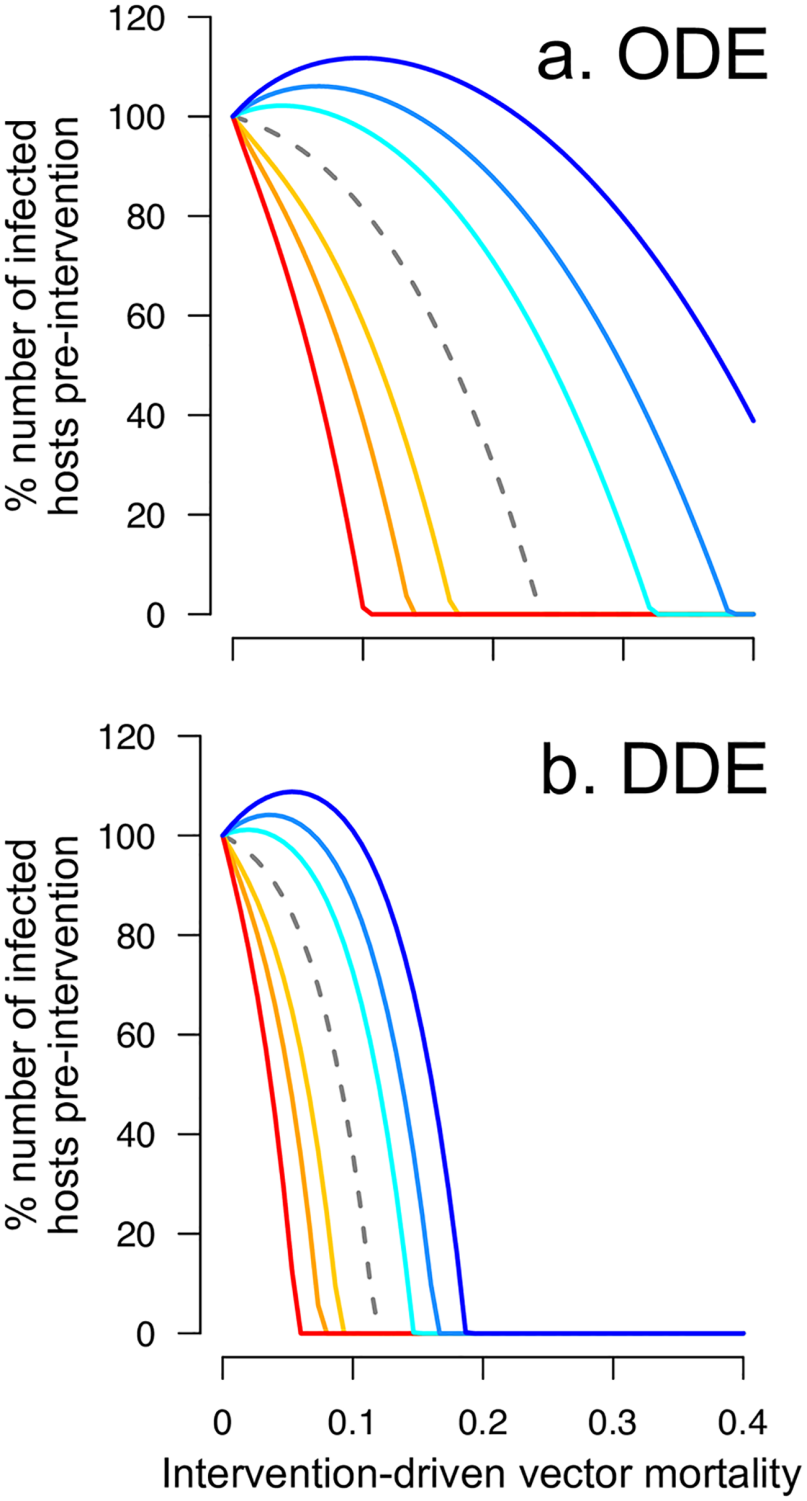
Both ordinary (ODE) and delay (DDE) differential equation models point to the possibility of an adverse consequence of moderate vector control interventions. Shown are the percentage of infection cases relative to the pre-intervention level predicted by (a) the ODE model and (b) the DDE model plotted against the intensity of intervention-driven vector mortality. The colour keys are as described in Fig 3.

This scenario presents a dilemma where vector control can successfully decrease the abundance of infectious vectors and symptomatic hosts (Fig 3a & 3b) while counterintuitively—and counterproductively—increasing the number of pre-sensitised infections and even the total abundance of infectious hosts (in both ODE and DDE models; Fig 4a & 4b, respectively). Further increases to vector mortality eventually outweigh the increase in the availability of pre-sensitised susceptible hosts, reducing the total number of infected hosts. If we assume the opposite effect of pre-exposure to vector saliva, i.e., pre-sensitisation increases the recovery rate either by promoting natural recovery or by exacerbating disease symptoms and ensuring earlier treatment, then the efficacy of vector control is enhanced by the pre-sensitisation effect (Fig 3 warm colours).

### Sensitivity to modelling assumptions and parameters

**Fig 5:**
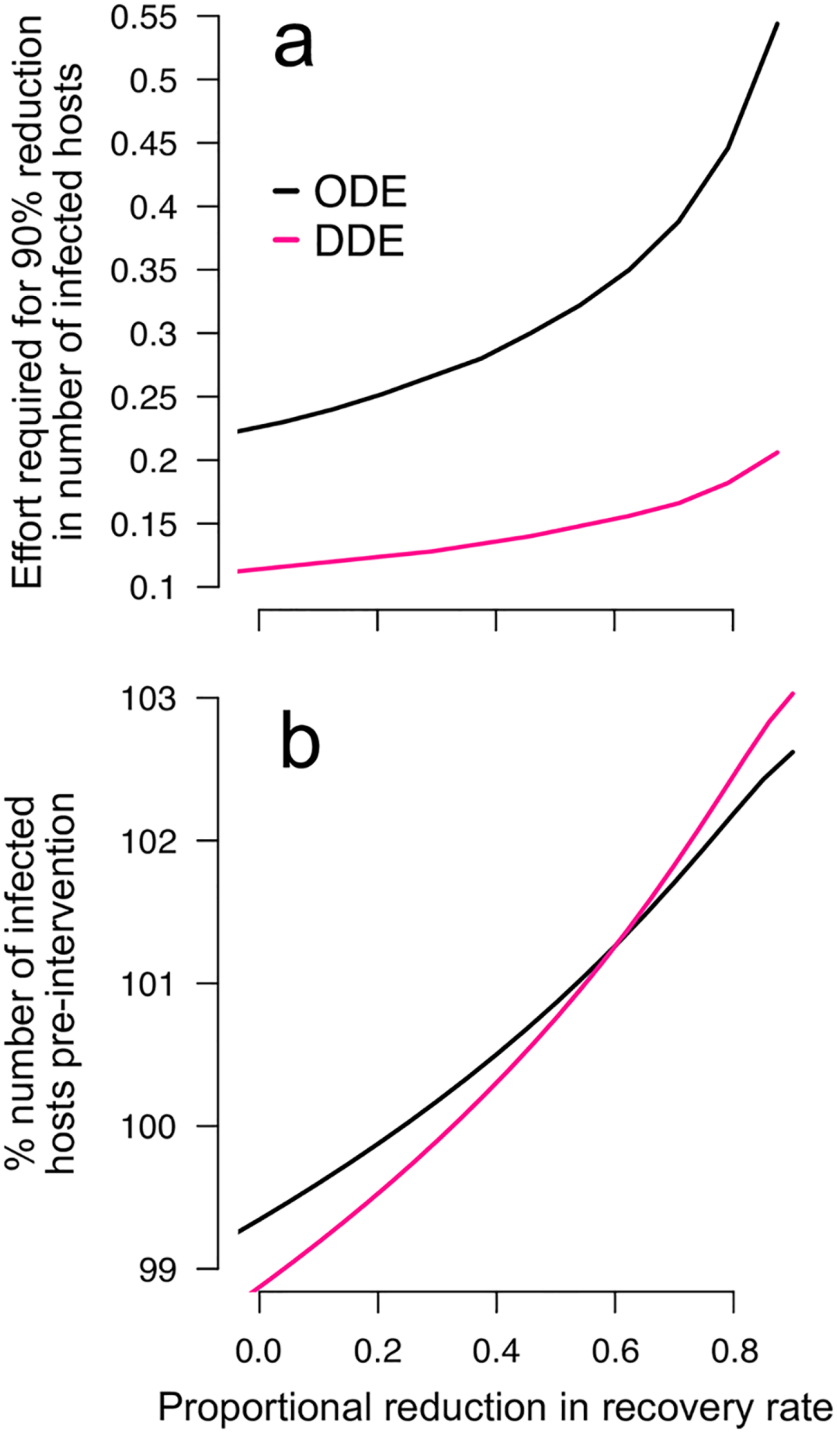
The severity and detectable signs of the adverse interaction between vector saliva pre-exposure and increased vector mortality are likely to be influenced by the effect of saliva pre-sensitisation in reducing the rate of recovery and the assumption about the variability in parasite development. Shown are (a) the level of intervention-driven vector mortality (“effort”) required to achieve a 90% reduction in the number of infected hosts and (b) the percentage of the pre-intervention number of infected hosts after a small reduction (25%) in vector lifespan due to vector control. The x-axis denotes the assumed effect of saliva pre-exposure on recovery as the proportional reduction in recovery rate.

Our model predicts that a moderate increase in vector mortality can elevate infection cases due to the change in the ratio of non-infectious to infectious vectors. Because large variability in the EIP assumed by the ODE model is expected to support a higher level of parasite transmission (because more vectors will survive the EIP when it is sometimes short), the severity of the adverse effect might be influenced by the assumption about the distribution of the EIP. Thus, we simulated the DDE model in which we expect fewer infectious vectors because the EIP is assumed to be fixed in duration. We find that both models predict the same qualitative outcome that a moderate increase in vector mortality can lead to an increase in infection cases when pre-sensitisation prolongs the time to recovery (Fig 4). However, eliminating the variation in the EIP reduces the parameter space over which control might be expected to increase the total number of infected hosts. Furthermore, the DDE model predicts that a lower intensity of control is needed than in the ODE model to achieve a 90% reduction in the number of infected hosts—a global target by 2030 set by WHO in the fight against malaria [46] (Fig 5a). Both models, nonetheless, predict that the intervention-driven vector mortality required to achieve this goal increases steeply if saliva pre-sensitisation decreases the rate of host recovery. It is noteworthy that the DDE model predicts that the initial response to vector control (e.g., the number of infected hosts after a small reduction in vector lifespan) can be exaggerated compared to the ODE model depending on how strongly saliva pre-sensitisation reduces the recovery rate (Fig 5b). Therefore, the adverse effect may occur regardless of the assumption about variability in parasite development, but the severity of the effect will likely change depending on the variability in the EIP.

Returning to the ODE model, we explored the parameter sensitivity in order to identify factors that influence the adverse interaction between vector saliva pre-sensitisation and interventions targeting vector survival. First, the unintended increase in pre-sensitised infected hosts is augmented when parameters associated with the vector population are favourable for parasite transmission, i.e., when vectors are born at a high rate, parasite incubation in the vector is fast, or the vector biting rate is high (*b*, *σ*_*V*_ and *ϕ*_*V*_, respectively; S3 Appendix). Second, experimental evidence from a rodent model of leishmaniasis suggests that the immunological effects of pre-exposure to vector saliva wane over time [47]; we find that if immune pre-sensitisation wanes fast enough, vector control is not predicted to increase the abundance of infected hosts (S3 Appendix, *θ*_*H*′_). Third, and counterintuitively, the vector control-driven increase in pre-sensitised infections is most pronounced when the probability of pre-sensitisation upon contact is low (but non-zero; S3 Appendix, *P*_*HV*_). At high probability, the number of pre-sensitised hosts is already high in the absence of vector control interventions, and increasing vector mortality through control decreases the force of infection more than it increases the likelihood of pre-exposure to vector saliva. Whereas at low probability, the increasing availability of susceptible pre-sensitised hosts due to control can outweigh the decreasing force of infection. Fourth, the increase in the pre-sensitised infections can be minimised by increasing the recovery rate of naïve, infected hosts, which in turn limits the overall parasite transmission (S3 Appendix, *γ*_*H*_). In addition, the vector control-mediated increase in pre-sensitised infections is minimal when immune memory is long lasting, because immune-waning provides a source of susceptible hosts that helps sustain parasite transmission S3 Appendix, *τ*_*H*_). Finally, understanding the contribution of pre-sensitised hosts to parasite transmission is the key to estimating the impact of saliva pre-sensitisation on epidemiology: our model predicts that the adverse consequence of vector control can be avoided if the probability of transmission to and from pre-sensitised hosts is considerably smaller than for naïve hosts (S3 Appendix, *T*_*H*′*V*_ and *T*_*VH*′_).

## Discussion

Prior exposure of mammalian hosts to uninfected vector bites can alter host immune responses against a variety of parasites [11]. While this three-way interaction between hosts, vectors, and parasites has been shown to influence progression of a wide range of vector-borne diseases [11, 12], the detailed biology of this interaction remains an active area of research and the epidemiological consequences are not obvious. Therefore, mapping the possible epidemiological implications relies on mathematical models [48]. Using a generic model of vector-borne diseases, we have shown that interventions targeting adult vector survival may increase the number of hosts that are pre-exposed to vector saliva. When pre-sensitisation mitigates disease symptoms as reported for *Leishmania*, *Plasmodium* and West Nile virus [10, 11, 19] and, as a consequence, prolongs the time to recovery through clinical interventions, we predict that a moderate increase in vector mortality can increase the infection cases in the host population. Alternatively, if immune pre-sensitisation leads to more rapid clearance of infection, we find that increasing vector mortality rates may achieve greater than expected disease control.

If the sole effect of a disease intervention is a modest increase in vector mortality, then our model predicts it could actually increase the public health burden when pre-sensitisation leads to milder and, ultimately, untreated infections. One might ask if an increase in sub-clinical infections is truly a concern for public health. The answer depends on patient health and transmission potential of those hosts. Evidence from a range of vector-borne diseases suggests that asymptomatic or sub-clinical patients suffer substantial viability and reproductive costs associated with carrying parasites [49–53]. From a population perspective, sub-clinical infections maintain a reservoir of active transmission, which may seed periodic outbreaks [54, 55]. Indeed, our analysis of *R*_0_ confirms that vector saliva pre-exposure can facilitate disease outbreaks under the assumption that the immunological effect of pre-exposure prolongs the duration of infection (S1 Appendix).

Immune sensitisation triggered by pre-exposure to vector saliva has been hypothesised to involve both antibody-based and cell-mediated immunity [5] with the two arms of immunity exhibiting distinct functional roles. For example, the suggested role of anti-salivary antibodies is to neutralise salivary proteins [5], which would otherwise facilitate parasite infection [11]. In contrast, cell-mediated responses at the site of an uninfectious bite are thought to hinder future nearby parasite infection as collateral damage [5]. The relative importance of different arms of immunity, and whether they lead to different disease outcomes and parasite transmission remain open questions [5, 56]. However, with the majority of experimental studies reporting quantitative effects (e.g., changes in parasite density and lesion size), it is unclear whether pre-sensitisation affects the probability of initial parasite establishment. Therefore, there is little direct evidence to suggest that immune sensitisation triggered by pre-exposure to vector saliva is powerful enough to offer complete protection from infection. On the other hand, the consensus finding from leishmaniasis, that pre-exposure leads to disease mitigation [11], is consistent with the notion that pre-exposed patients are more likely to remain sub-clinical [56]. Further understanding of the immunological pathways involved in pre-sensitisation, as well as the success of parasites in pre-sensitised hosts (e.g., probability of establishment and transmitted parasite density) and the clinical outcome for the host (e.g., recovery and mortality) will open possibilities for predictions of epidemiological patterns.

Theories on vaccination and immune priming predict that mechanisms that improve host health (and hence prolong infection), but do not block transmission can increase disease prevalence in host populations [57, 58]. Supporting the theory, a recent experimental study demonstrated that vaccination of chickens against Marek’s disease virus leads to increased cumulative transmission of highly virulent viral strains because vaccination prolongs infection without preventing transmission [59]. These previous findings as well as the results presented here invite careful examination of clinical consequences associated with saliva-derived vaccines (reviewed in [18]) so that vaccination achieves a desired goal both clinically and epidemiologically over a long timescale.

Our study suggests that caution is warranted in interpreting empirical estimates regarding the impact of interventions. First, the efficacy of vector control is rarely reported at the human population level [32]; instead, studies often rely on signals from vector populations. Our model shows that an optimistic signal from the vector population—reduced vector abundance and reduced proportion of infected individuals in the vector population—can coincide with increasing sub-clinical cases and an overall increase in infection cases in the host population. Therefore, estimates of infection cases in the host population are crucial for assessing intervention efficacy. Second, parasite prevalence in a population is often inferred from the number of clinical cases, which is inevitably limited to symptomatic patients who seek treatment. Our results demonstrate that even when vector control decreases the force of infection, it may simultaneously increase the abundance of infected hosts pre-sensitised by vector saliva whose infections may go undetected due to milder symptoms. In light of growing evidence that sub-clinical hosts are infectious to arthropod vectors [60, 61], our results reinforce the need for active surveillance in order to accurately estimate parasite transmission in a given population.

Our findings highlight the importance of both thorough spraying programmes and integrated vector management approaches, especially when control is expected to achieve only moderate increases in vector mortality. Fortunately, vector control tools are rarely used in isolation; rather, multiple intervention approaches are integrated to target different components of the parasite transmission cycle [62]. Our parameter sensitivity analysis underscores the importance of integrated vector management by showing that parameters associated with conventional methods for combatting vector-borne diseases—reduced vector birth rate (e.g., removing standing water where female vectors lay their eggs), reduced vector-biting rate (e.g., use of bednets), and increased recovery of symptomatic hosts (e.g., treatment efficacy)—all help limit the risk that increased vector mortality will elevate the number of pre-sensitised infections. Finally, predictions about the consequences of integrated control might require better quantitative knowledge about the underlying density-dependent processes in the vector population. For example, increased adult mortality should lead to reduced egg laying, which may ultimately reduce adult recruitment [35]. However, increased adult mortality could also relax larval competition, leading to greater subsequent adult recruitment [37]. Which of these effects prevails will likely be system- (and possibly environment-) specific, but will likely impact epidemiological outcomes.

### Conclusion

We demonstrated that pre-exposure to vector saliva alters epidemiological outcomes in a manner that could be positive or negative to public health. It is currently not possible to make precise epidemiological predictions due to the gaps of detailed knowledge about the effect of pre-sensitisation on clinical outcomes and parasite transmission, and more generally in vector ecology. Filling in these gaps will be crucial for delineating the potentially negative interaction between pre-exposure to saliva and vector control, and for deploying saliva-based vaccines effectively in the future. Our work underscores the importance of considering the interplay among vector biology, host immunity, and control measures so that the combined effect of interventions yield desirable disease control outcomes.

## Supporting information

S1 Appendix Disease-free equilibrium and calculating *R*_0_. (PDF)

S2 Appendix Transient dynamics with and without control. (PDF)

S3 Appendix Parameter sensitivity. (PDF)
