# Supplementary material for: Epidemiological consequences of immune sensitisation by pre-exposure to vector saliva
Source: PLoS Negl Trop Dis. 2017 Oct 9;11(10):e0005956. doi: 10.1371/journal.pntd.0005956 (PMC5648264; doi:10.1371/journal.pntd.0005956)
Supplement: S1 Appendix — (PDF) [file pntd.0005956.s001.pdf]

S1 Appendix: Disease-free equilibrium and calculating R_0

In the ODE model, when a vector population is free of parasites, the change in the number of vectors, V_S, is described simply by:

$$\frac{dV_S(t)}{dt} = \phi_V - \mu_V V_S(t) \tag{S1}$$

where ϕ_V and μ_V are the rates of vector birth and death respectively. By setting $\frac{dV_S(t)}{dt}$ equal to zero, we obtain the disease-free vector equilibrium, i.e. $\widehat{V_S} = \frac{\phi_V}{\mu_V}$.

Similarly, in the absence of parasites, the change in the number of naïve susceptible, H_S, susceptible hosts is described as

$$\frac{dH_S(t)}{dt} = -rP_{HV}V_S(t)H_S(t) + \theta_{H'}(H_T - H_S(t)) \tag{S2}$$

where r is the rate at which a host gets bitten by a vector (defined as $\frac{b}{H_T}$ where b is the biting rate per vector and H_T is the constant total host population size), P_{HV} is the probability of pre-sensitisation per biting event, and $\theta_{H'}$ is the rate of loss of the saliva pre-exposure effect. Note that $H_T - H_S(t)$ is the number of susceptible hosts that are pre-exposed to vector saliva. By setting $\frac{dH_S(t)}{dt}$ equal to zero, we obtain the disease-free susceptible host equilibrium,

$$\widehat{H_S} = \frac{\theta_{H'}{H_T}^2}{bP_{HV}\widehat{V_S} + \theta_{H'}H_T}, \tag{S3}$$

and the equilibrium number of pre-sensitised susceptible hosts, $\widehat{H'_S}$, is simply $H_T - \widehat{H_S}$.

The risk of an epidemic outbreak is conventionally expressed as the basic reproductive number, R_0, which is the number of secondary infections produced when one infected individual is introduced to an entirely susceptible population of hosts. Here we calculate R_0 using the next generation method [1], which is a general approach to calculate R_0 for infection cycles involving multiple infected compartments as the dominant eigenvalue of the next generation matrix (refer to Heffernan et al. [2] for an accessible overview of this approach). Following the notation of Heffernan et al. [2], the infection matrix, F and the transition matrix, V for our ODE model (eq. 1 main text) are described as follows:

$$F = \left\{ \begin{array}{cccc} 0 & 0 & rT_{VH}V_S & rT_{VH'}V_S \\ 0 & 0 & 0 & 0 \\ 0 & rT_{HV}H_S & 0 & 0 \\ 0 & rT_{H'V}H'_S & 0 & 0 \end{array} \right\}, \tag{S4}$$

$$V = \left\{ \begin{array}{cccc} \mu_V + \sigma_V & 0 & 0 & 0 \\ -\sigma_V & \mu_V & 0 & 0 \\ 0 & 0 & \gamma_H & 0 \\ 0 & 0 & 0 & \gamma_{H'} \end{array} \right\}. \tag{S5}$$

The next generation matrix is then calculated as the product of the infection matrix and the inverse of the transition matrix, FV^{-1} as follows:

$$FV^{-1} = \left\{ \begin{array}{cccc} 0 & 0 & \frac{rT_{VH}V_S}{\gamma_H} & \frac{rT_{VH'}V_S}{\gamma_{H'}} \\ 0 & 0 & 0 & 0 \\ \frac{\sigma_V rT_{HV}H_S}{\mu_V^2+\mu_V\sigma_V} & \frac{rT_{HV}H_S}{\mu_V} & 0 & 0 \\ \frac{\sigma_V rT_{H'V}H'_S}{\mu_V^2+\mu_V\sigma_V} & \frac{rT_{H'V}H'_S}{\mu_V} & 0 & 0 \end{array} \right\}. \tag{S6}$$

Then the dominant eigenvalue of the matrix FV^{-1} gives R_0:

$$R_0 = \frac{r\sqrt{V_S}\sqrt{\sigma_V}\sqrt{H_S T_{HV} T_{VH}\gamma_{H'} + H'_S T_{H'V} T_{VH'}\gamma_{H'}}}{\sqrt{\gamma_H}\sqrt{\gamma_{H'}}\sqrt{\mu_V(\mu_V+\sigma_V)}}. \tag{S7}$$

The effect of saliva-induced pre-sensitisation on the chance of a disease outbreak can be assessed by taking the partial derivative of R_0 with respect to the probability of successful pre-sensitisation upon contact, i.e., P_{HV}. While the full expression of $\frac{\partial R_0}{\partial P_{HV}}$ is rather large and not shown here, we find the following proportional relationship:

$$\frac{\partial R_0}{\partial P_{HV}} \propto T_{H'V}T_{VH'}\gamma_H - T_{HV}T_{VH}\gamma_{H'}. \tag{S8}$$

The sign of this expression determines the directional influence of saliva pre-sensitisation probability on R_0: when positive, pre-sensitisation increases the chance of an outbreak and when negative it decreases the same chance. This term can be rearranged to give a condition for when pre-sensitisation increases R_0:

$$\frac{T_{H'V}T_{VH'}}{T_{HV}T_{VH}} > \frac{\gamma_{H'}}{\gamma_H}. \tag{S9}$$

This expression simply tells us that pre-sensitisation facilitates disease outbreaks if the ratio of the product of the transmission probabilities (i.e., vector to host and host to vector transmission) of pre-sensitised hosts to naïve hosts is greater than the relative recovery rate of pre-sensitised hosts to

naïve hosts. Such a scenario is foreseeable if pre-exposure to saliva leads to milder infections that are rarely treated, and susceptibility and infectivity of pre-sensitised hosts remain sufficiently high.

References

[1] Diekmann O, Heesterbeek JAP, Metz JA. On the definition and the computation of the basic reproduction ratio R0 in models for infectious diseases in heterogeneous populations. J Math Biol. 1990;28(4):365–382.

[2] Heffernan J, Smith R, Wahl L. Perspectives on the basic reproductive ratio. J R Soc Interface. 2005;2(4):281–293.
