# Supplementary material for: Epidemiological consequences of immune sensitisation by pre-exposure to vector saliva
Source: PLoS Negl Trop Dis. 2017 Oct 9;11(10):e0005956. doi: 10.1371/journal.pntd.0005956 (PMC5648264; doi:10.1371/journal.pntd.0005956)
Supplement: S2 Appendix — (PDF) [file pntd.0005956.s002.pdf]

S2 Appendix: Transient dynamics with and without control

We simulated the transient infection dynamics of the ODE model to investigate the short term consequences of vector control in the presence of saliva pre-sensitisation (Fig. A1). In the short term, vector control suppresses the peak number of infections for both naïve and pre-sensitised hosts (i.e., Fig. A1a & b) and the proportion of vectors that are infectious (Fig. A1d). However, vector control can increase the equilibrium abundance of pre-sensitised infected hosts and even the overall infection cases when pre-exposure to vector saliva prolongs the time to recovery (Fig. A1b & c).

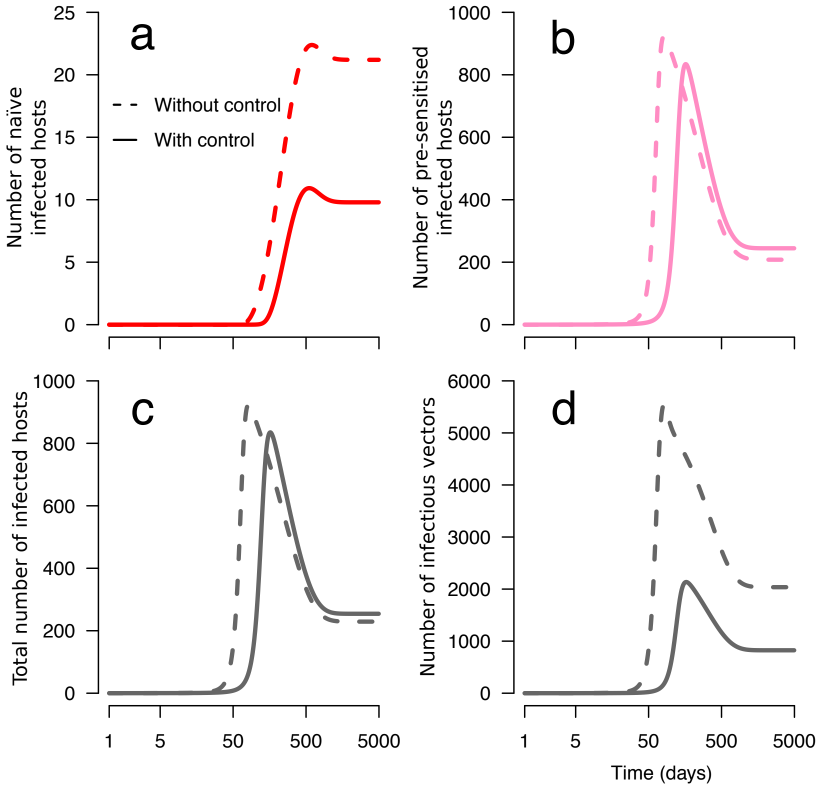

Figure A1: A moderate increase in vector mortality reduces the peak, but can increase the equilibrium, number of infections in hosts. The solid lines represent a moderate increase in vector mortality through control efforts ($\mu_V = 7^{-1}$ per day compared to the baseline mortality of 14^{-1} presented as dashed lines). Shown are (a) the number of naïve infected hosts (H_I) and (b) pre-sensitised infected hosts (H_I'), (c) number of all hosts that are infected, regardless of pre-sensitisation status and (d) number of infectious vectors. The x-axes are on a $\log_{10}$ scale. Disease introduction and an intervention targeting vector survival take place simultaneously at the beginning of the simulations. The recovery rate of the pre-sensitised hosts is set to be $^1/_5 th$ that of naïve hosts, who recover at a rate of 60^{-1} per day.
