# Supplementary material for: Epidemiological consequences of immune sensitisation by pre-exposure to vector saliva
Source: PLoS Negl Trop Dis. 2017 Oct 9;11(10):e0005956. doi: 10.1371/journal.pntd.0005956 (PMC5648264; doi:10.1371/journal.pntd.0005956)
Supplement: S3 Appendix — (PDF) [file pntd.0005956.s003.pdf]

S3 Appendix: Parameter sensitivity

We explored the parameter sensitivity of the result that pre-sensitised infections can increase in response to vector control targeting vector survival using the ODE model (Fig. A2). The change in the number of pre-sensitised infections due to control is driven by the change in 1) the availability of pre-sensitised susceptible hosts, and 2) the force of infection. Therefore, the increase in the number of pre-sensitised infections is most pronounced in parameter spaces where the change in the availability of pre-sensitised susceptible hosts due to control is large (e.g., low rate of loss of saliva pre-exposure effect, $\theta_{H'}$) and the force of infection is high (e.g., high vector biting rate, b).

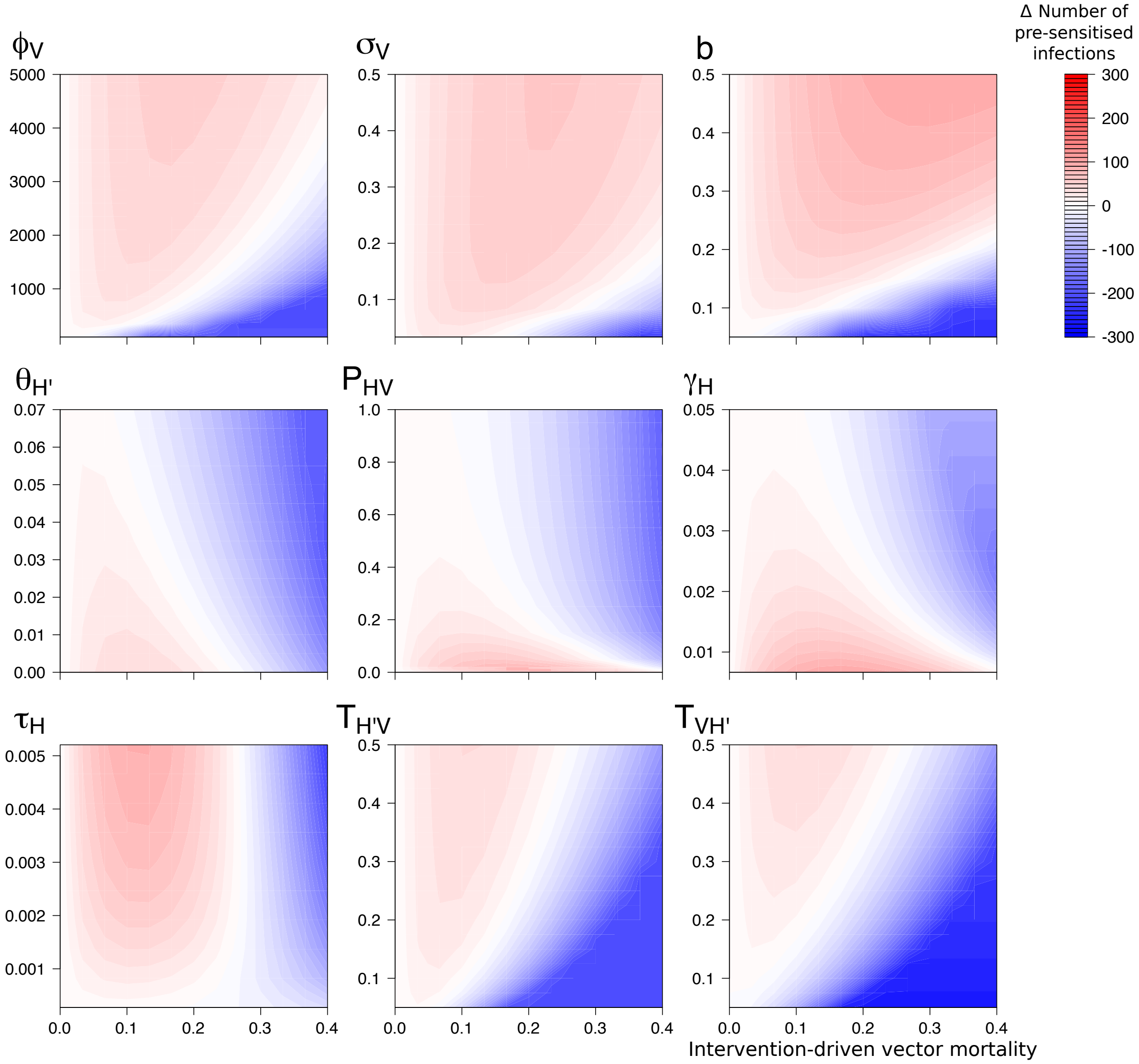

Figure A2: Parameter sensitivity. Contour plots show the change of the number of pre-sensitised infections as a function of the rate of intervention-driven vector mortality (μ_V; x-axis) and a parameter of interest labelled on each y-axis. Parameters explored in the ODE model are vector birth rate (ϕ_V), parasite incubation rate in the vector (σ_V), vector biting rate (b), rate of loss of saliva pre-exposure effect ($\theta_{H'}$), pre-sensitisation probability upon contact (P_{HV}), recovery rate of naïve hosts (γ_H), rate of loss of acquired immunity (τ_H), transmission probability from naïve host to vector (T_{VH}) and transmission probability from pre-sensitised host to vector ($T_{VH'}$). Areas in red show increasing number of pre-sensitised infections in response to vector control, while areas in blue decreasing pre-sensitised infections. (The y-axes delineates the baseline conditions of no vector control and only natural vector mortality). The parameter ranges explored are given in Table 1 in the main text. Parameters that are not explicitly varied in each panel are set as defaults in Table 1.
